# Supplementary material for: Genomic and immune landscape Of metastatic pheochromocytoma and paraganglioma
Source: Nat Commun. 2023 Feb 28;14:1122. doi: 10.1038/s41467-023-36769-6 (PMC9975198; doi:10.1038/s41467-023-36769-6)
Supplement: Supplementary file 1 — Supplementary Information [file 41467_2023_36769_MOESM1_ESM.pdf]

SUPPLEMENTARY INFORMATION

GENOMIC AND IMMUNE LANDSCAPE OF METASTATIC PHEOCHROMOCYTOMA AND PARAGANGLIOMA

Bruna Calsina[1#], Elena Piñeiro-Yáñez[2,*], Ángel M Martínez-Montes[1,*], Eduardo Caleiras[3], Ángel Fernández-Sanromán[1], María Monteagudo[1], Rafael Torres-Pérez[1,4], Coral Fustero-Torre[2], Marta Pulgarín-Alfaro[1], Eduardo Gil[1], Rocío Letón[1], Scherezade Jiménez[5], Santiago García-Martín[2], Maria Carmen Martin[6], Juan María Roldán-Romero[1], Javier Lanillos[1], Sara Mellid[1], María Santos[1], Alberto Díaz-Talavera[1,7], Ángeles Rubio[8], Patricia González[3], Barbara Hernando[9], Nicole Bechmann[10], Margo Dona[11], María Calatayud[12], Sonsoles Guadalix[12], Cristina Álvarez-Escolá[13], Rita M Regojo[14], Javier Aller[15], Maria Isabel del Olmo-Garcia[16], Adrià López-Fernández[17], Stephanie M J Fliedner[18], Elena Rapizzi[19], Martin Fassnacht[20,21], Felix Beuschlein[22,23], Marcus Quinkler[24], Rodrigo A Toledo[25,26], Massimo Mannelli[19], Henri J Timmers[11], Graeme Eisenhofer[10,27], Sandra Rodríguez-Perales[6], Orlando Domínguez[8], Geoffrey Macintyre[9], Maria Currás-Freixes[1,28], Cristina Rodríguez-Antona[1,7], Alberto Cascón[1,7], Luis J Leandro-García[1], Cristina Montero-Conde[1,7], Giovanna Roncador[5], Juan Fernando García-García[29], Karel Pacak[30], Fátima Al-Shahrour[2], Mercedes Robledo[1,7#]

[1] Hereditary Endocrine Cancer Group, Human Cancer Genetics Program, Spanish National Cancer Research Centre (CNIO), Madrid, Spain
[2] Bioinformatics Unit, Structural Biology Program, Spanish National Cancer Research Centre (CNIO), Madrid, Spain
[3] Histopathology Core Unit, Biotechnology Program, Spanish National Cancer Research Centre (CNIO), Madrid, Spain
[4] Bioinformatics for Genomics and Proteomics, National Centre for Biotechnology (CNB-CSIC), Madrid, Spain
[5] Monoclonal Antibodies Core Unit, Biotechnology Program, Spanish National Cancer Research Centre (CNIO), Madrid, Spain
[6] Molecular Cytogenetics and Genome Engineering Group, Human Cancer Genetics Program, Spanish National Cancer Research Centre (CNIO), Madrid, Spain
[7] Centro de Investigación Biomédica en Red de Enfermedades Raras (CIBERER), Institute of Health Carlos III (ISCIII), Madrid, Spain
[8] Genomics Core Unit, Biotechnology Program, Spanish National Cancer Research Centre (CNIO), Madrid, Spain
[9] Computational Oncology Group, Structural Biology Program, Spanish National Cancer Research Centre (CNIO), Madrid, Spain
[10] Institute of Clinical Chemistry and Laboratory Medicine, University Hospital Carl Gustav Carus, Technische Universität Dresden, Dresden, Germany
[11] Department of Internal Medicine, Radboud University Medical Centre, Nijmegen, The Netherlands
[12] Department of Endocrinology, 12 de Octubre University Hospital, Madrid, Spain
[13] Department of Endocrinology, La Paz University Hospital, Madrid, Spain
[14] Department of Pathology, La Paz University Hospital, Madrid, Spain
[15] Department of Endocrinology, Puerta de Hierro University Hospital, Madrid, Spain
[16] Department of Endocrinology and Nutrition, University Hospital La Fe, Valencia, Spain
[17] Department of Medical Oncology, Vall d'Hebrón Hospital, Barcelona, Spain
[18] Neuroendocrine Oncology and Metabolism, Medical Department I, Center of Brain, Behavior, and Metabolism, University Medical Center Schleswig-Holstein Lübeck, Lübeck, Germany
[19] Department of Experimental and Clinical Biomedical Sciences, University of Florence, Florence, Italy
[20] Department of Internal Medicine I, Division of Endocrinology and Diabetes, University Hospital Würzburg, University of Würzburg, Würzburg, Germany
[21] Comprehensive Cancer Center Mainfranken, University of Würzburg, Würzburg, Germany
[22] Medizinische Klinik und Poliklinik IV, Klinikum der Universität München, Munich, Germany
[23] Klinik für Endokrinologie Diabetologie und Klinische Ernährung, Universitätsspital Zürich (USZ) und Universität Zürich (UZH), Zürich, Switzerland
[24] Endocrinology in Charlottenburg Stuttgarter Platz 1, Berlin, Germany
[25] Gastrointestinal and Endocrine Tumors, Vall d'Hebron Institute of Oncology (VHIO), Vall d'Hebron Barcelona Hospital Campus, Barcelona, Spain
[26] Centro de Investigación Biomédica en Red de Cáncer (CIBERONC), Institute of Health Carlos III (ISCIII), Madrid, Spain.
[27] Department of Medicine III, University Hospital Carl Gustav Carus, Technische Universität Dresden, Dresden, Germany
[28] Department of Endocrinology, Clínica Universidad de Navarra, Madrid, Spain
[29] Department of Pathology, MD Anderson Cancer Center, Madrid, Spain
[30] Section of Medical Neuroendocrinology, *Eunice Kennedy Shriver* National Institute of Child Health and Human Development, National Institutes of Health, Bethesda, Maryland, USA
* These authors contributed equally.

# Corresponding authors
Bruna Calsina – email: bcalsina@cnio.es
Mercedes Robledo – email: mrobledo@cnio.es

Supplementary Table 1. Clinical characteristics of patients with pheochromocytomas and paragangliomas included in CNIO series. Related to Fig. 1.

**Patient's Clinical Characteristics**	**WES series (n = 61)** % (n)	**RNA-Seq series (n = 104)** % (n)
**Primary tumor localization**		
PCC	32.8% (20)	37.5% (39)
PGL	31.1% (19)	36.6% (38)
bilateral PCC	3.3% (2)	3.8% (4)
multiple PGL	11.5% (7)	3.8% (4)
PCC+PGL	13.1% (8)	10.6% (11)
NA	8.2% (5)	7.7% (8)
**Sex**		
Female	49.2% (30)	50% (52)
Male	41% (25)	46.1% (48)
NA	9.8% (6)	3.9% (4)
**Age at initial diagnosis of PCC/PGL; median (range) in years**	38.5 (10-82)	42 (10-82)
**Clinical behavior**		
Non-metastatic disease	3.3% (2)	41.4% (43)
Aggressive disease	4.9% (3)	9.6% (10)
Metastatic disease	91.8% (56)	49% (51)

**Tissue Sample Characteristics**	**WES series (n = 87)** % (n)	**RNA-Seq series (n = 118)** % (n)
**Tumor type**		
Primary PPGL tumor	64.4% (56)	82.2% (97)
Second Primary PPGL tumor	4.6% (4)	0.9% (1)
Primary PPGL relapse	4.6% (4)	2.5% (3)
Metastases	26.4% (23)	11.9% (14)
Normal adrenal medulla	0% (0)	2.5% (3)
**Anatomic localization of primary tumor sequenced**		
PCC	50% (32)	55.4% (56)
Abdominal PGL	6.2% (4)	13.9% (14)
Thoracic PGL	6.2% (4)	4% (4)
Head & Neck PGL	9.4% (6)	3% (3)
Retroperitoneal PGL	16.1% (9)	8.9% (9)
Mediastinal PGL	3.1% (2)	1% (1)
Paravesical PGL	1.6% (1)	1% (1)
unknown location of PGL	9.4% (6)	12.9% (13)

Supplementary Fig. 1

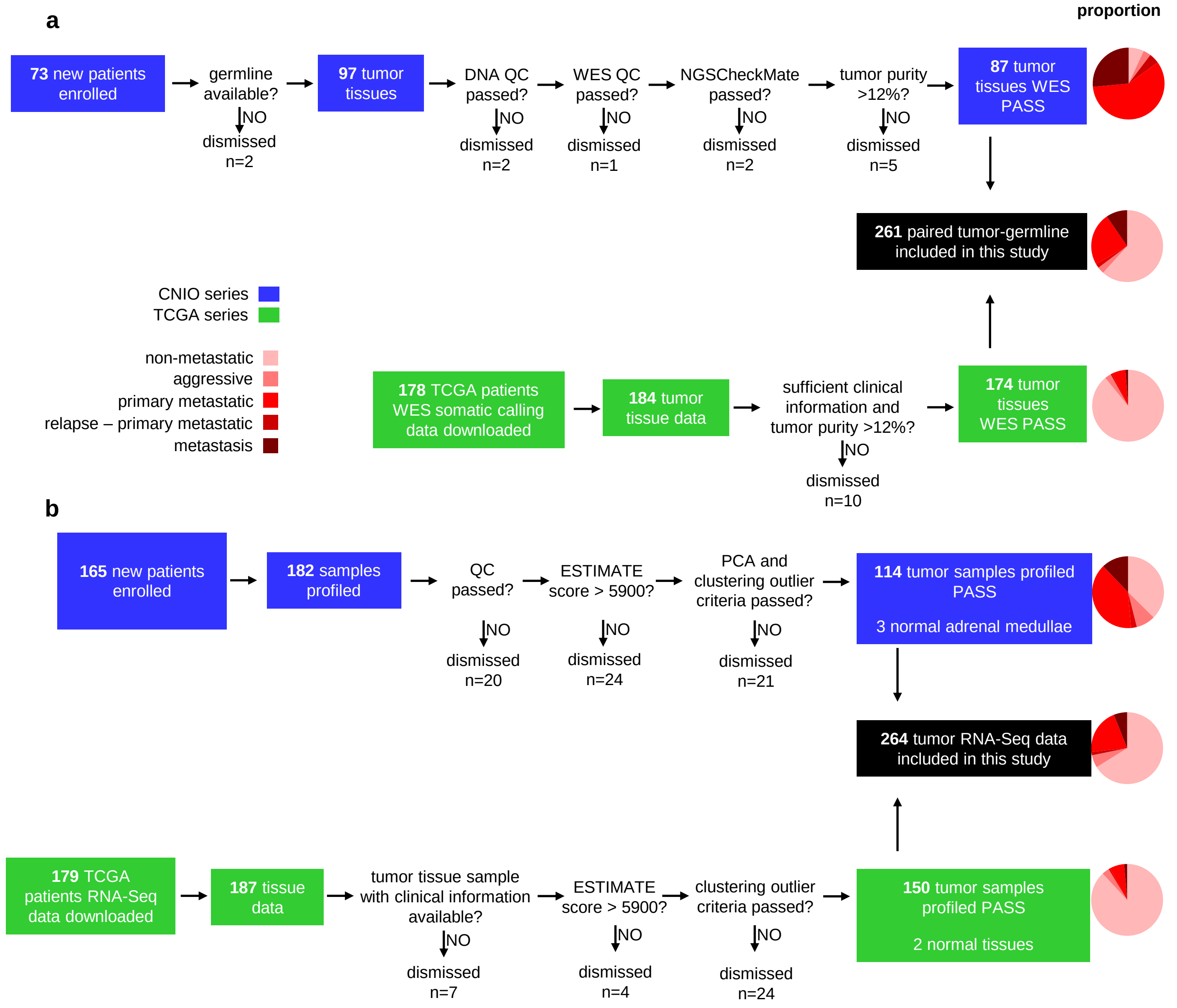

Supplementary Fig. 1. Sample workflow for WES and RNA-Seq analyses. (a) A total of 73 patients were enrolled between January 2017 and April 2019 (blue boxes – CNIO cohort) and included in WES study. For 2.7% of patients, no germline material was obtained. Sequencing was performed in 97 tumors from 71 patients. However, 2 samples failed during DNA isolation and 1 sample failed WES quality control analysis. In two out of the remaining 94 samples, tumor and germline data did not pair (tested using WES BAMs with *NGSCheckMate*) and 5 had a tumor purity lower than 12% (based on WES data using *FACETS*), and were excluded from the analysis. In total, 87 pairs of tumor and normal samples were included in this study, representing the 90% of the CNIO collected cohort. From the TCGA series (green boxes), somatic variant calling data from 174 tumors (95%) was included in the series; 10 tumors were dismissed due to insufficient clinical data available or tumor purity lower than 12% (estimated using *FACETS*). In total, 261 paired tumor-germline data was used for the analysis: 61.7% were non-metastatic primary tumors, 23.7% were metastatic primary tumors, 3.1% were primary tumors from patients with aggressive disease, 1.9% were relapses from metastatic primary tumors, and 9.6% were metastases. **(b)** For the RNA-Seq study, in the CNIO series (blue boxes), 165 patients were enrolled between January 2017 and August 2019. These corresponded to 182 tissue samples, but 20 tumors did not pass the raw RNA-Seq data quality control, 24 had a low tumor purity (estimated by *ESTIMATE* tumor content purity; score>5900) and 21 were outliers in the PCA, consensus and hierarchical clustering analyses, so they were dismissed. In total, 114 tumor samples and 3 normal adrenal medullae were analyzed in the CNIO RNA-Seq study. From the TCGA series (green boxes), RNA-Seq raw counts were obtained from 187 tissues from 179 patients. In total, 35 samples were removed from the series either because they did not have associated clinical information, the tumor purity estimation was low (based on re-analysis using *ESTIMATE,* score>5900), or clustering analysis identified them as outliers. In total, 150 tumor samples and 2 normal tissues from TCGA were merged together with the 117 samples of the CNIO cohort in a unique matrix. Batch effects were corrected with *ComBat*. The final RNA-Seq series is composed by 66.4% non-metastatic primary tumors, 20.5% of metastatic primary tumors, 5.6% primary tumors from patients with aggressive disease, 1.5% relapses from metastatic primary tumors, and 6% metastases.

Supplementary Fig. 2

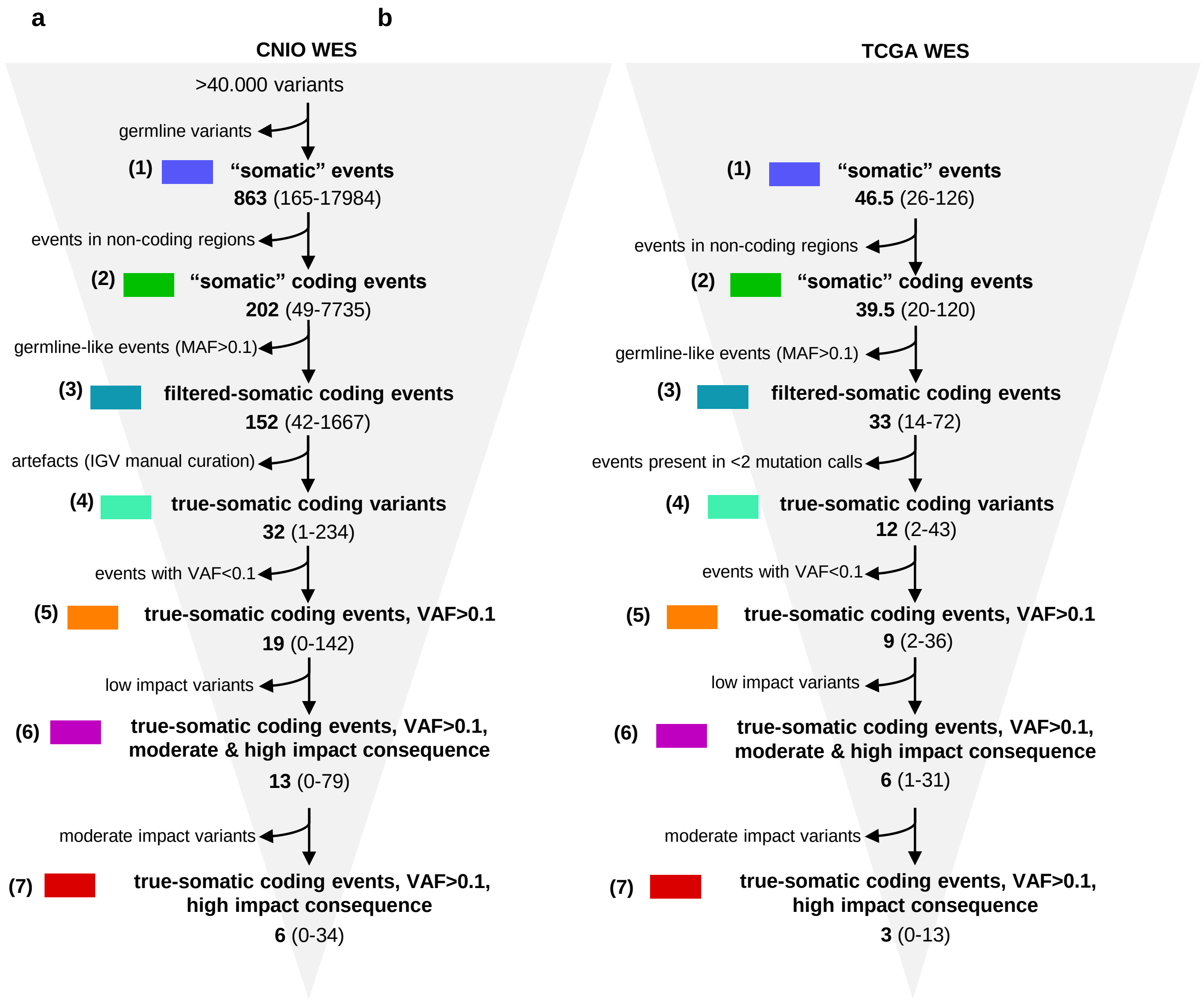

Supplementary Fig. 2. Pipeline for variant filtering and candidate gene prioritization. Numbers in bold under each category stand for the median number of events found in the 87 WES from the CNIO cohort **(a)** or 174 WES from the TCGA cohort **(b)**; range is shown in brackets. Variants included in categories (5), (6) and (7) are the ones used in all subsequent analyses. VAF: Variant allele fraction. Color code is the same as in Fig. 2.

Supplementary Fig. 3

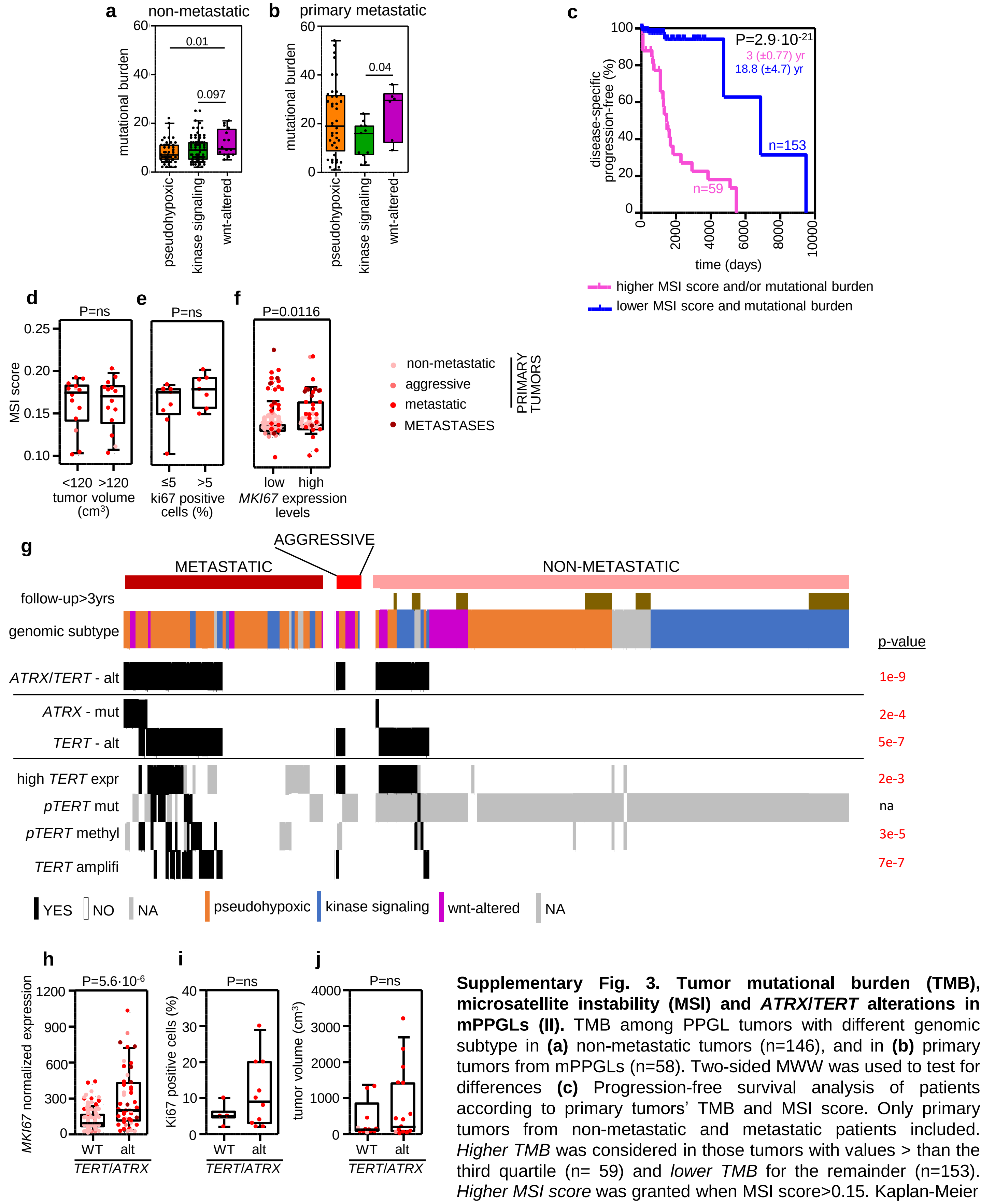

Supplementary Fig. 3. Tumor mutational burden (TMB), microsatellite instability (MSI) and *ATRX/TERT* alterations in mPPGLs (II). TMB among PPGL tumors with different genomic subtype in **(a)** non-metastatic tumors (n=146), and in **(b)** primary tumors from mPPGLs (n=58). Two-sided MWW was used to test for differences **(c)** Progression-free survival analysis of patients according to primary tumors' TMB and MSI score. Only primary tumors from non-metastatic and metastatic patients included. *Higher TMB* was considered in those tumors with values > than the third quartile (n= 59) and *lower TMB* for the remainder (n=153). *Higher MSI score* was granted when MSI score>0.15. Kaplan-Meier plot of time to progression (time between the first PPGL diagnosis and the first documented metastasis) is shown together with the P-value calculated using a log-rank test. Median progression time (± standard error) of each group is depicted in the corresponding color. Patients without evidence of metastases were censored at the date of the last follow-up. n: number of patients. MSI score according to **(d)** tumor volume (cm^3) (n=28), **(e)** % Ki67 positive cells (n=16) and **(f)** *MKI67* mRNA expression (n=191). Volumes and % Ki67 positive cells data, when available, were extracted from the pathological anatomy reports received with each specimen. *High MKI67 mRNA expression* indicates levels above the 3rd quartile value of the whole cohort and *low MKI67 mRNA low expression* when levels were beneath the 3rd quartile value. **(g)** Landscape of *ATRX* and *TERT* alterations in the whole cohort. Two-sided Fisher's exact test was used to test for differences between metastatic (as in Figure 2I) and non-metastatic groups. *ATRX* mutations (Supplementary Table 2), and *TERT* alterations (which includes high *TERT* expression, *TERT* amplification, *TERT* promoter (*pTERT*) mutations and hypermethylation) were considered. **(h)** *MKI67* expression, **(i)** Ki67 positive cells (%) and **(j)** tumor volume (cm^3) in *ATRX/TERT*-wild-type (WT) and in *ATRX/TERT*-altered tumors (n=191, n=16 and n=28, respectively for h, i and j). Two-sided MWW was used to test for differences. For all box-plots in this figure: the median value is marked, and Tukey whiskers are represented.

Supplementary Table 2. ATRX mutations

patient ID	Primary	Relapse	Metastasis
**METASTATIC**			
**#1**	c.1094del; p.Asn365ThrfsTer2<br>frameshift_variant<br>VAF=0.38	–	c.1094del; p.Asn365ThrfsTer2<br>frameshift_variant<br>VAF=0.45
**#2**	c.1485del; p.Lys495Asnfs*19<br>frameshift_variant<br>VAF=0.39	–	NA
**#3**	c.6875del; p.Pro2292Glnfs*28<br>frameshift_variant<br>VAF=0.71	c.6875del;<br>p.Pro2292Glnfs*28<br>frameshift_variant<br>VAF=0.73	c.6875del; p.Pro2292Glnfs*28<br>frameshift_variant<br>VAF=0.65
**#4**	c.1441G>T; p.Glu481Ter<br>stop_gained<br>VAF=0.76	–	metastasis 1: c.1501G>T;<br>p.Glu501Ter<br>stop_gained<br>VAF=0.67<br>metastasis 2: c.1441G>T;<br>p.Glu481Ter<br>stop_gained<br>VAF=0.38
**#5**	c.2914_2917del;<br>p.Asp972Argfs*30<br>frameshift_variant<br>VAF=0.79	–	NA
**#6**	c.3622dup; p.Ile1208AsnfsTer4<br>frameshift_variant<br>VAF=0.87	–	NA
**#7**	NA	–	c.1493_1496del;<br>p.Arg498LysfsTer15<br>frameshift_variant<br>VAF=0.4
**#8**	NA	–	c.5268_5272+7del<br>splice_donor_variant<br>VAF=0.36
**NON-METASTATIC**			
**#9**	c.5657G>A; p.Pro1886Leu<br>missense_variant - deleterious<br>VAF=0.37	–	–

NA: non-available; – : no-occurrence; VAF: variant allele fraction

Supplementary Fig. 4

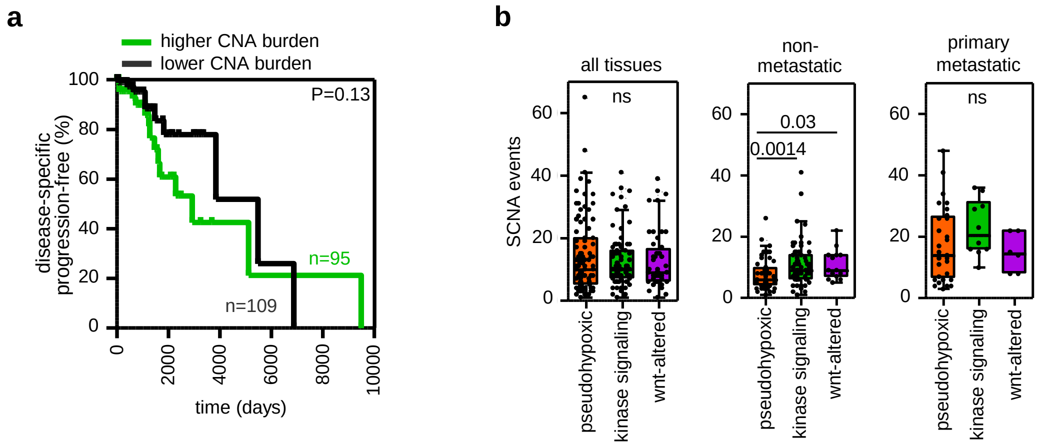

Supplementary Fig. 4. Somatic copy number alterations (SCNA) profile in mPPGLs (II). The data included correspond to 234 tumor-normal pairs from the CNIO and TCGA cohorts. **(a)** Progression-free survival analysis of patients according to primary tumors' SCNA burden (based on the number of events), respectively. Only primary tumors from non-metastatic and metastatic patients included. *Higher SCNA burden* indicates tumors with values > than the median (n=95), while lower SCNA burden < than the median (n=109). Kaplan-Meier plot of time to progression (time between the first PPGL diagnosis and the first documented metastasis) is shown together with the P-value calculated using the log-rank test. Median progression time (± standard error) of each group is depicted in the corresponding color. Patients without evidence of metastases were censored at the date of the last follow-up. n: number of patients. **(b)** SCNA events across PPGL tumors per genomic subtype including all tissues available (n=213), only non-metastatic tumors (n=136), or only metastatic primary tumors (n=50) with SCNA events data available. Two-sided MWW was used to test for differences. For all box-plots in this figure: the median value is marked, and Tukey whiskers are represented.

Supplementary Fig. 5

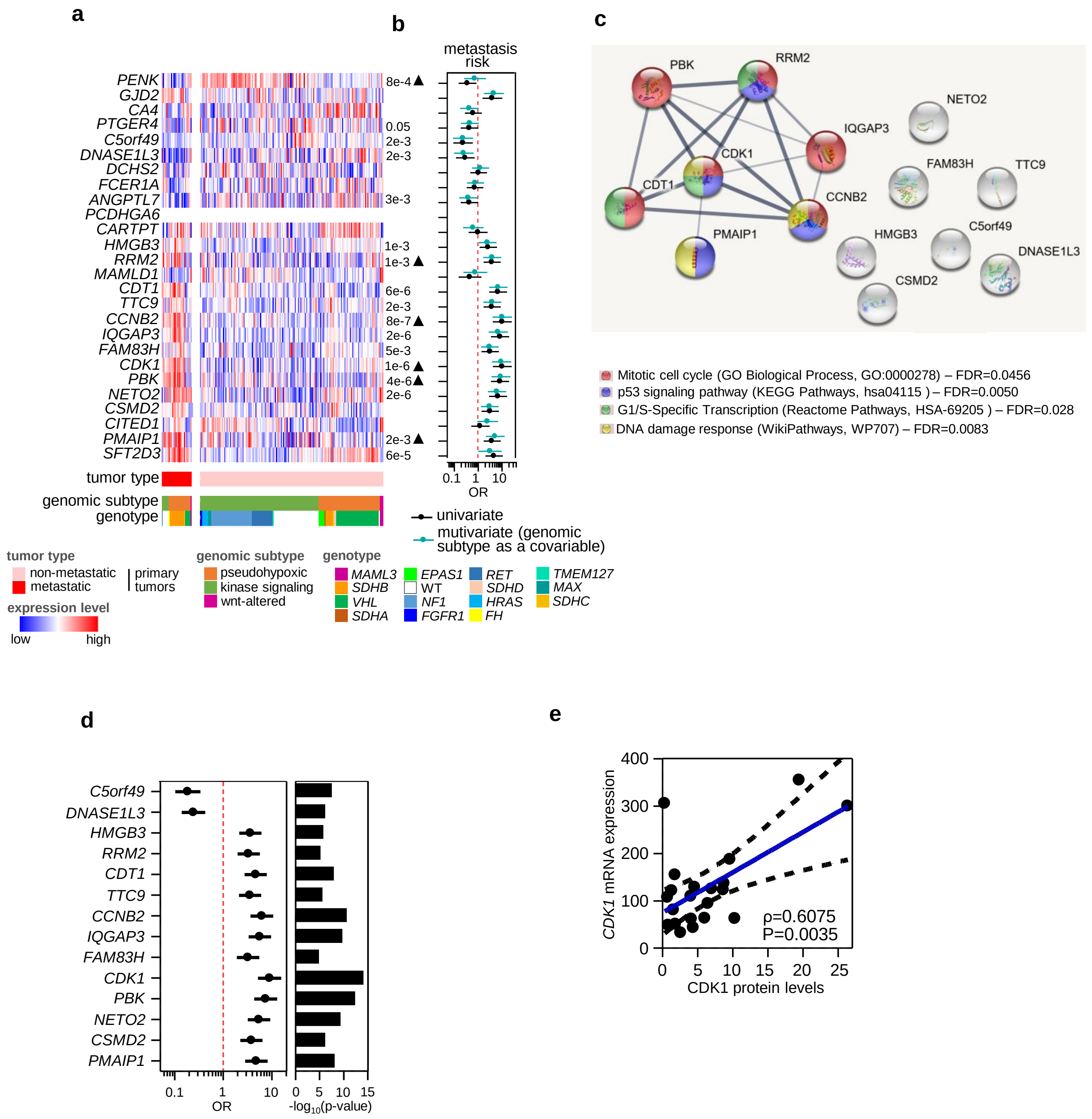

Supplementary Fig. 5. mPPGL transcriptomic profile (II). (a) Selected gene signature associated with mPPGL in an independent cohort (Burnichon et al., 2011). mRNA expression levels of the 27 selected genes, tumor behavior, genomic subtype and genotype are depicted in rows; primary tumors appear in columns. MWW two-sided test was applied to test for differences in expression between groups (metastatic and non-metastatic tumors), * : P<0.05 and ** : P<0.001. Only probes with > log2 fold-change between groups are shown. ▲- differential expressed genes in an independent DE analysis in this cohort. **(b)** Univariate (black) and multivariate (blue) logistic regression analysis of metastasis risk. Gene expression was dichotomized as follows: for down-regulated genes in mPPGLs, median expression was used as threshold (0 – below the median expression level; 1 – above the median expression level); for up-regulated genes in mPPGLs, high expression was considered for those with levels > than the 3rd quartile (0 – below the 3rd quartile threshold value; 1 – above the 3rd quartile threshold value). Multivariate analysis included as covariate genomic subtype. **(c)** String-db (https://string-db.org/) network view of the 15 genes forming the validated mPPGL signature. Network nodes represent proteins, and edges symbolize protein-protein interactions (line thickness indicates the strength of data support of that interaction). Minimum required interaction score was set to 0.4. Node color refers to the enriched gene set they belong to, as shown in the legend. Enrichment analysis was performed using the 'analysis' tool of String; FDR values shown are the ones calculated by String after correcting p-values for multiple testing using Benjamini-Hochberg procedure. **(d)** Combined multivariate logistic regression analysis of metastasis risk using data included in Fig. 4b,c. Gene expression was dichotomized as detailed before. Genomic subtype and series (this study and Burnichon's study) were used as covariates for the analysis. **(e)** Correlation between *CDK1* gene expression and protein levels measured by IHC in a subset of tumors with both data available (n=21). Two-sided Pearson's correlation coefficient is shown, P value and the 95% confidence band of the best-fit line.

Supplementary Fig. 6

a

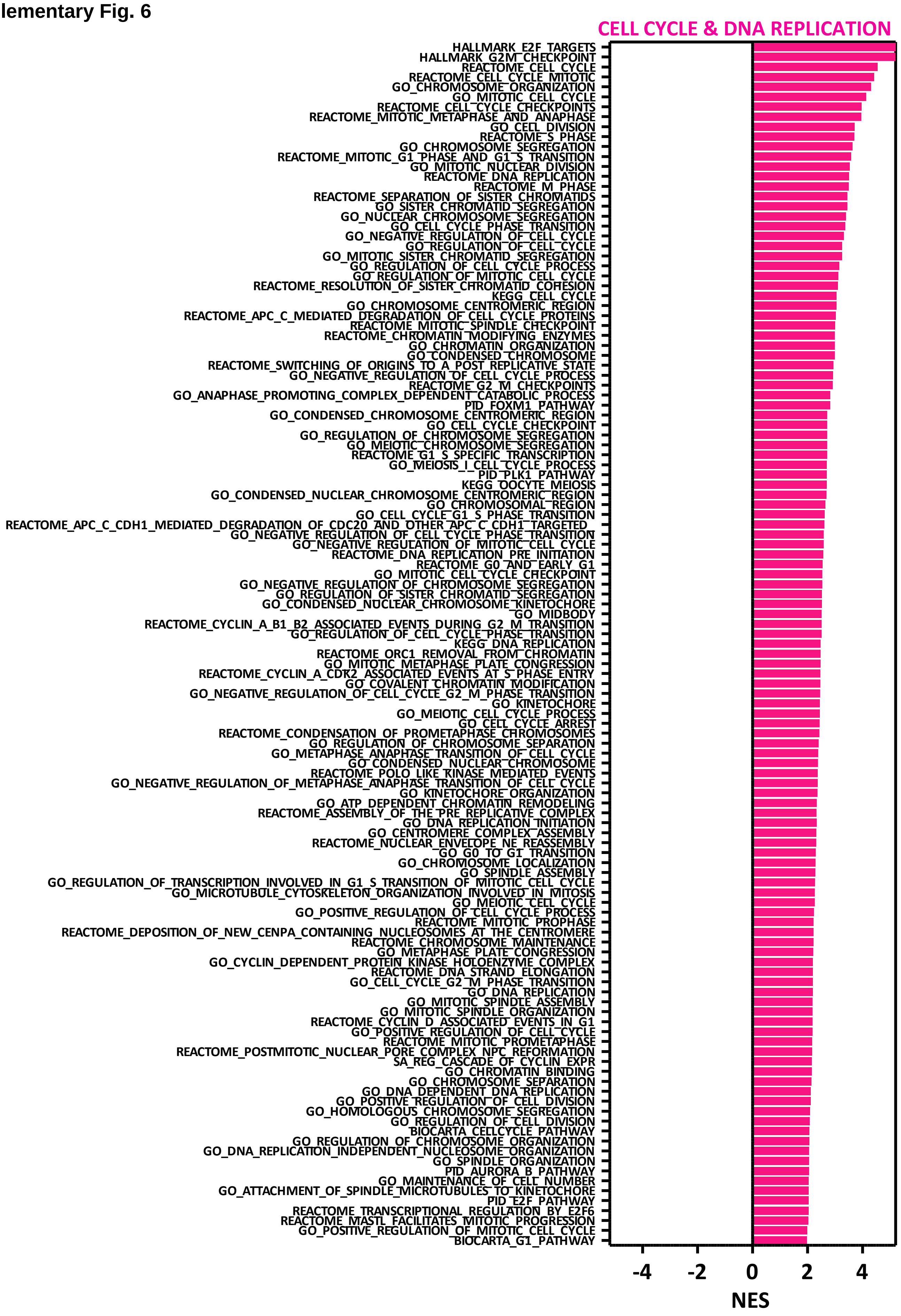

Supplementary Fig. 6. Breakdown of the biological processes into the gene sets identified by GSEA to be differentially represented in metastatic primary *versus* non-metastatic tumors (related to Fig. 4g). **(a)** cell cycle and DNA replication**, (b)** translation and ribosomes, **(c)** DNA repair and TP53 pathway, **(d)** ubiquitin and proteasome, **(e)** WNT pathway**, (f)** cilium, **(g)** Rho-GTPases, **(h)** ion transport, **(i)** GPCR, **(j)** circulatory system-vasculature development, **(k)** nervous system, **(l)** ECM organization, **(m)** cell adhesion and motility, **(n)** mitochondrial processes, and **(o)** immune response.

Supplementary Fig. 6

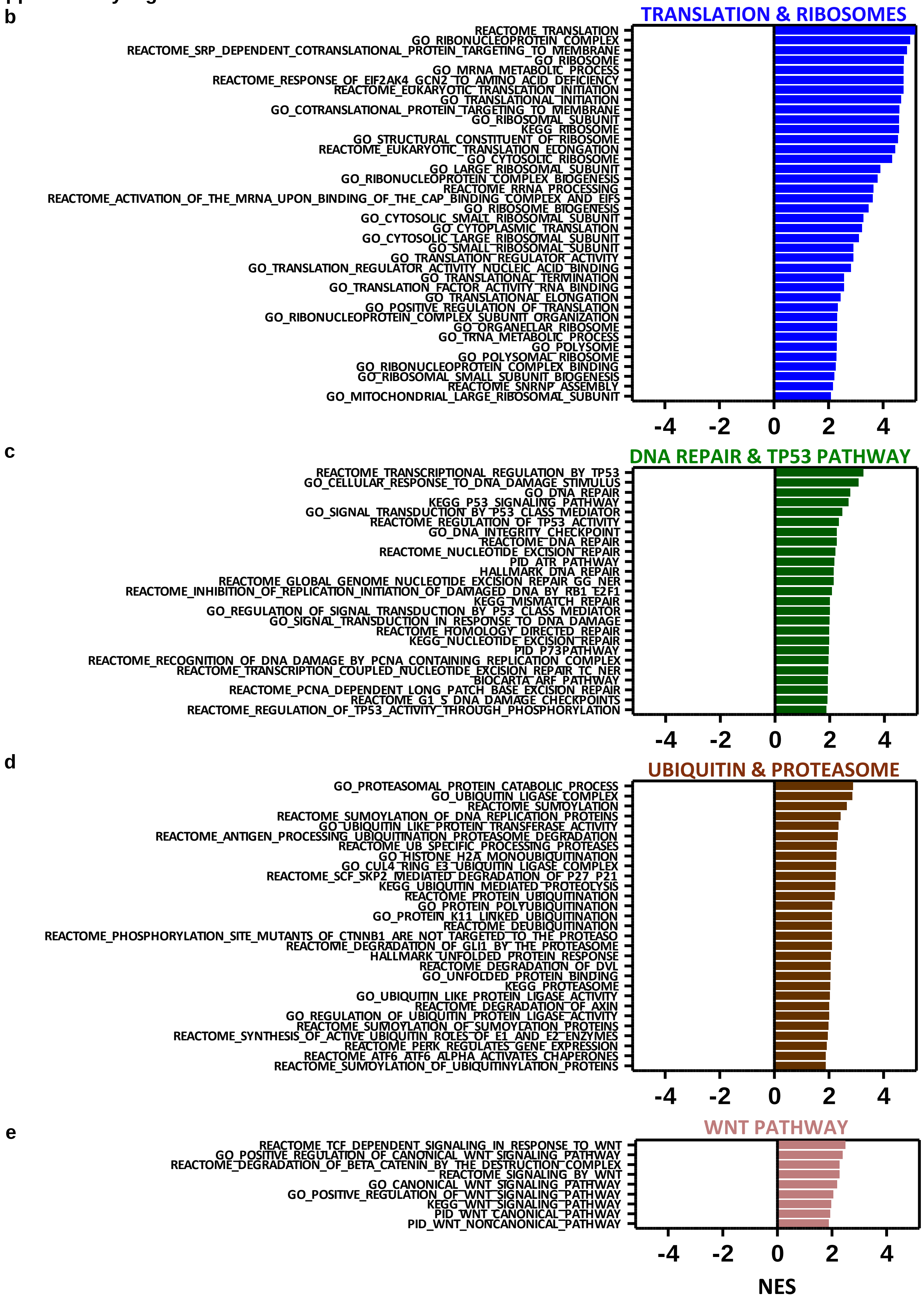

Supplementary Fig. 6

f

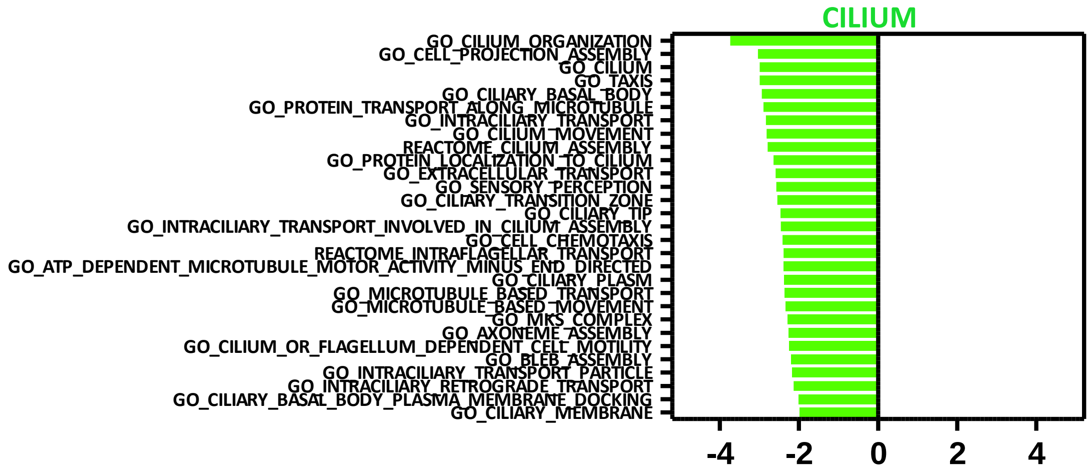

g

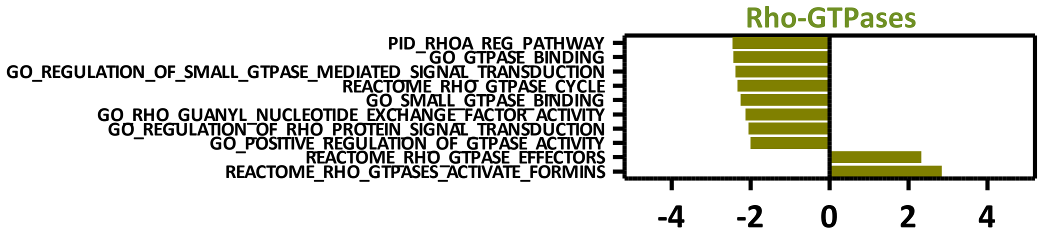

h

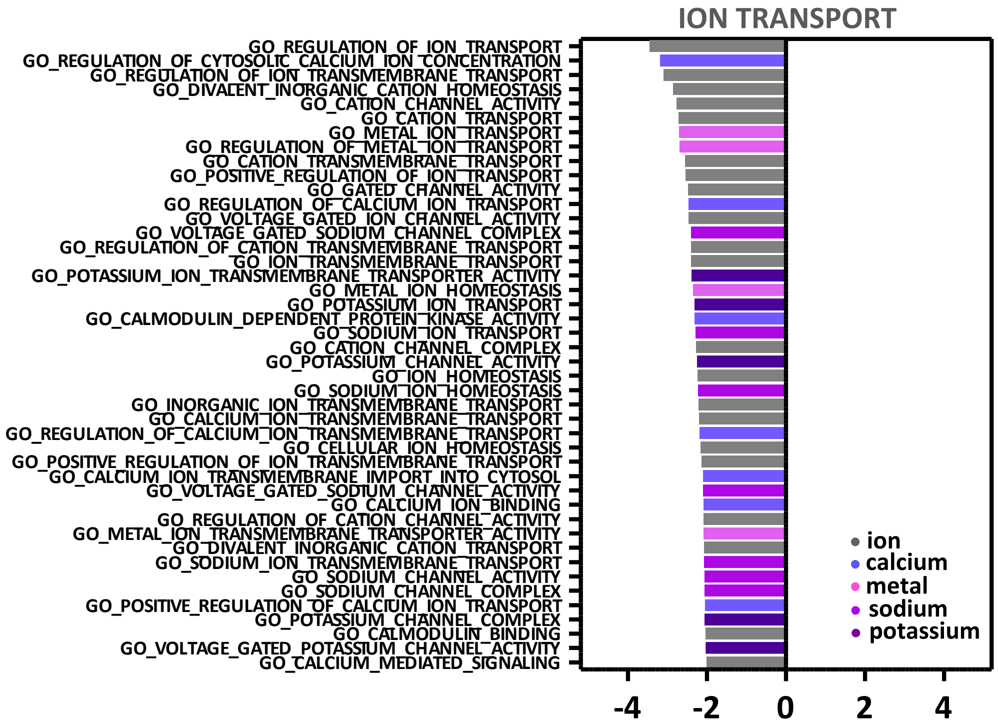

i

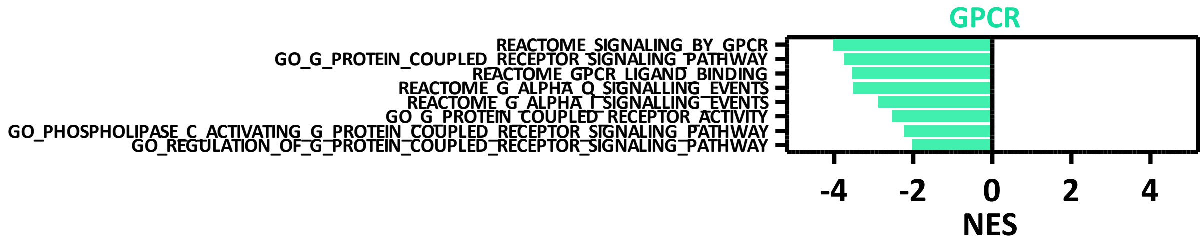

Supplementary Fig. 6

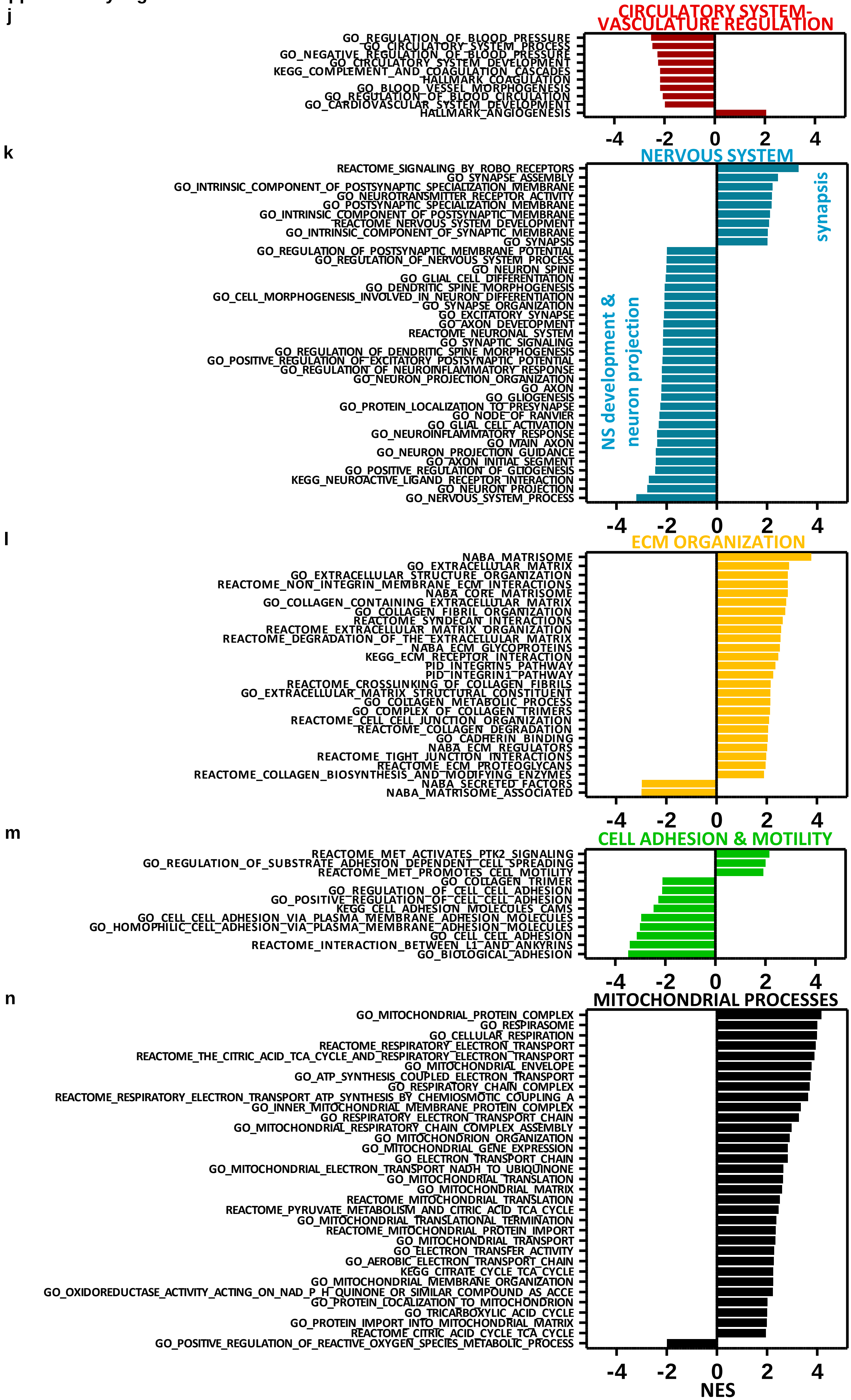

Supplementary Fig. 6

o

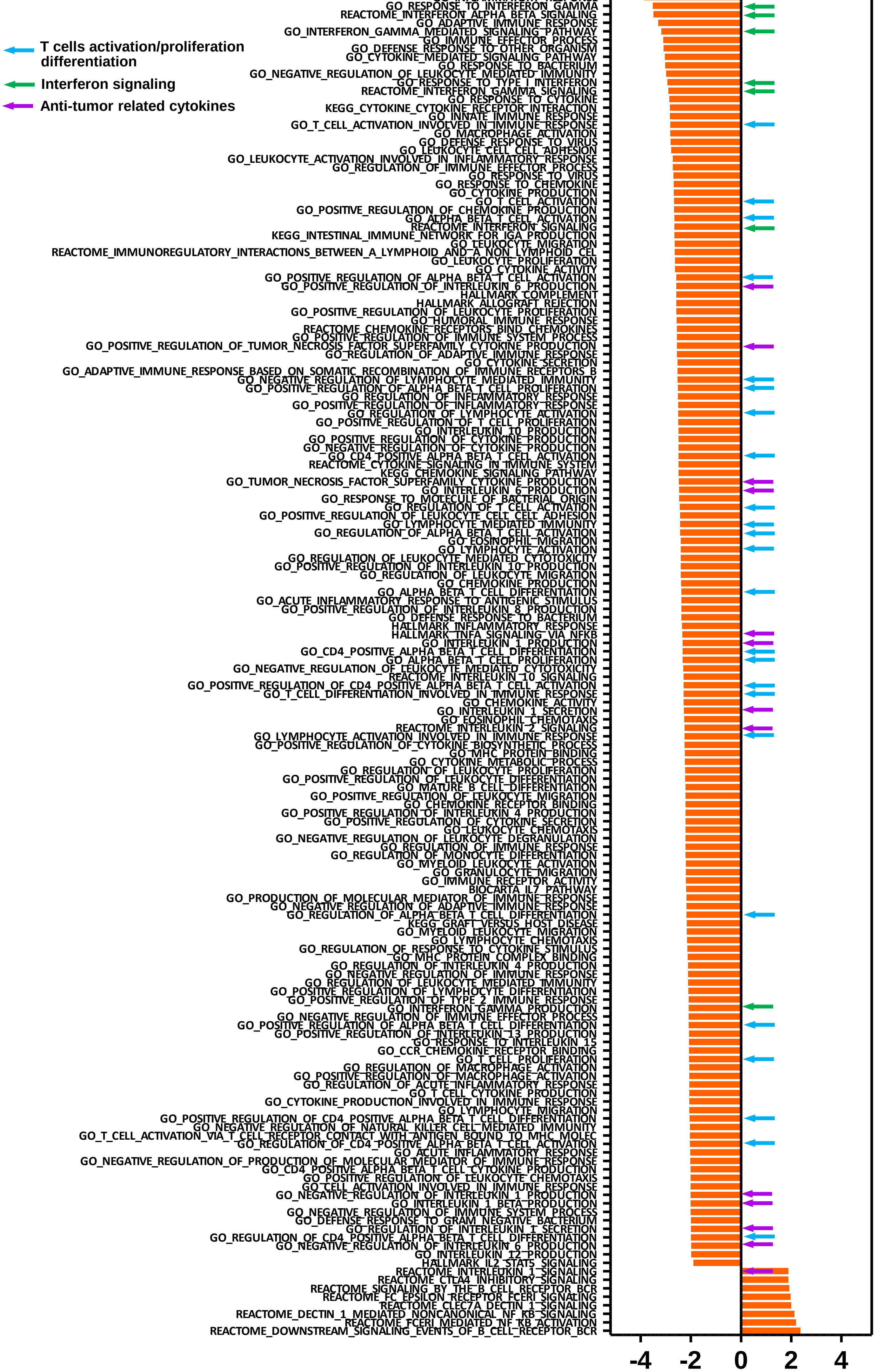

Supplementary Fig. 7

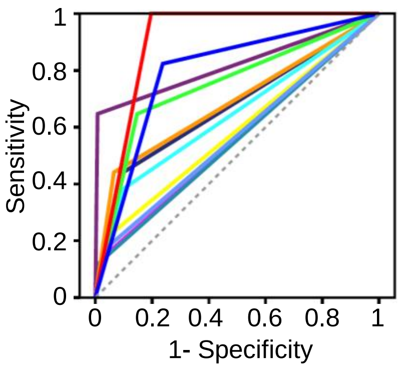

	AUC [95% CI]	P-value	Sensitivity	Specificity
ATRX-mut/ high MSI score/ high *CDK1* expr./ *MAML3*-fusion	0.902 [.855, .948]	8.58e-13	100	80.3
ATRX-mut/ *TERT*-alt/ Krebs Cycle gene-mut/ *MAML3*-fusion/ high *MIK67* expr.	0.793 [.706, .879]	1.83e-7	82.3	76.2
high MSI score	0.819 [.719, .920]	1.29e-8	64.7	99.2
high *CDK1* expr.	0.750 [.647, .852]	8.70e-6	64.7	85.2
high TMB	0.688 [.574, .801]	8.26e-4	44.1	93.4
Krebs Cycle gene-mut	0.671 [.558, .785]	2.27e-3	44.1	90.2
TERT-alt	0.642 [.527, .757]	1.15e-2	38.2	90.2
high *MIK67* expr.	0.587 [.470, .703]	0.12	20.6	96.7
chr 5 gains	0.568 [.452, .683]	0.23	17.6	95.9
MAML3-fusion	0.557 [.442, .672]	0.31	14.7	96.7
ATRX-mut	0.555 [.440, .670]	0.33	11.8	99.2

Supplementary Fig. 7. Receiver operating characteristic curve analysis showing the ability of the best classifier for mPPGL (*ATRX*-mut, high MSI score, high *CDK1* expression and MAML3-fusion), the ability of the classifier that includes the already known mPPGL markers (*ATRX*-mut, germline Krebs cycle mutations, *MAML3*-fusion and high *MIK67* expression), and the ability of each event independently. The AUC, 95%CI and sensitivity/specificity for the rest of classifiers is in Supplementary Data 1.

Supplementary Fig. 8

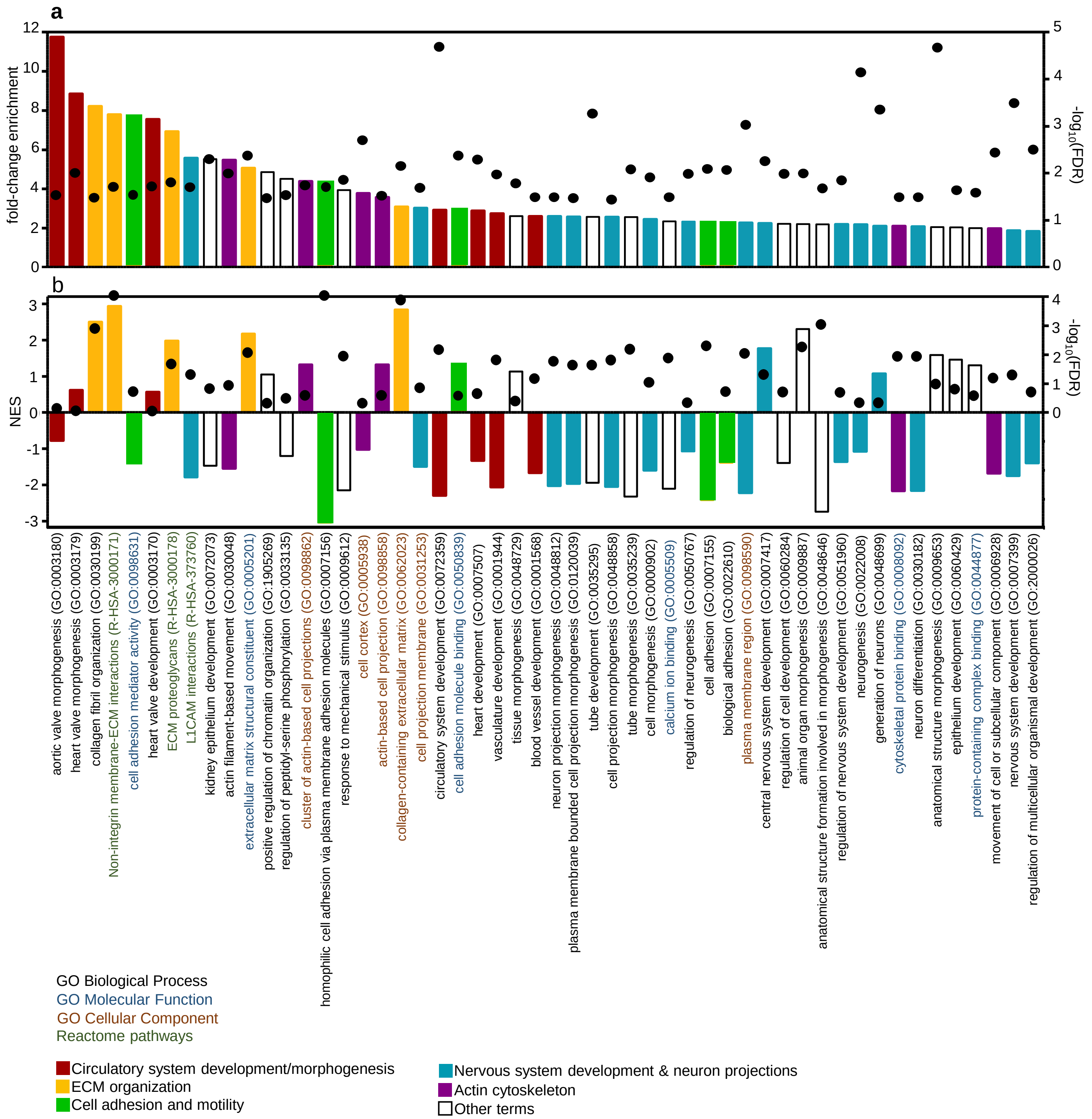

Supplementary Fig. 8. Functional enrichment analysis of mutated genes in mPPGLs. Related to Fig. 5. **(a)** Gene sets enriched in the list of 323 mutated genes in the metastatic primary tumors using GO enrichment analysis tool (http://geneontology.org/docs/go-enrichment-analysis/). Only those gene sets with a fold-change enrichment (FCe)>1.8 and FDR<0.05 by Fisher's Exact test were considered. Column height indicates FCe as specified in the left y-axis; black dots show -$\log_{10}$(FDR) as detailed in the right y-axis. **(b)** GSEA analysis of gene sets appeared in (A) using RNA-Seq series of metastatic *versus* non-metastatic primary tumors. Column height shows the normalized enrichment score (NES) as quantified in the left y-axis; black dots display -$\log_{10}$(FDR) as indicated in the right y-axis. The 52 gene sets identified are listed in the bottom of panels (A) and (B); the letters' font is related to the annotated data repository used (GO Biological Process, GO Molecular Function, GO Cellular Component or Reactome pathways) as indicated in the legend. The terms related to 'circulatory system development/morphogenesis' are shown in red, those related to 'ECM organization' in yellow, those involved in 'cell adhesion and motility' in green, those associated with 'NS development and neuron projection' in blue, and those related to 'actin cytoskeleton' in purple.

Supplementary Fig. 9

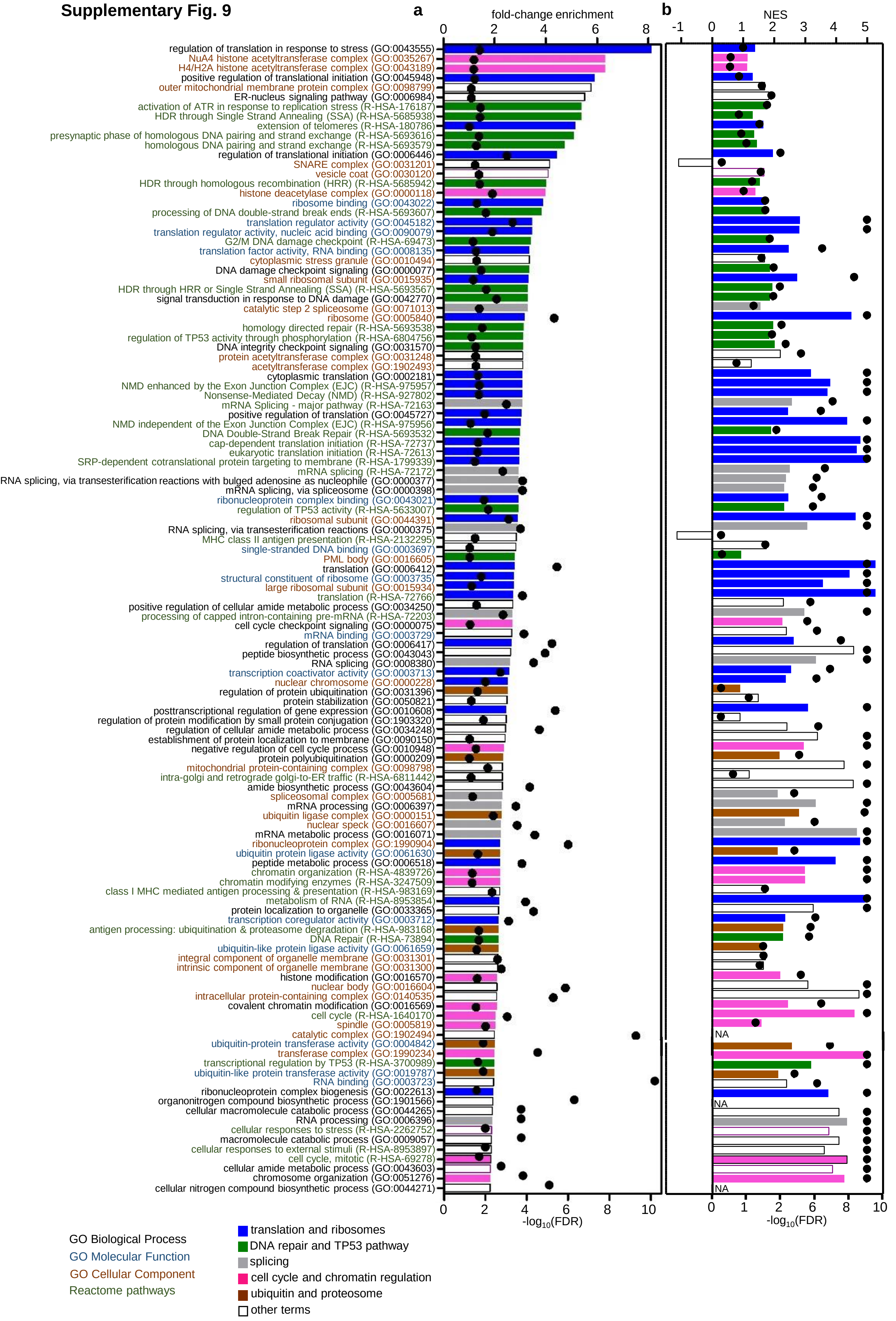

Supplementary Fig. 9. Functional enrichment analysis of CN-altered genes in mPPGLs. Related to Fig. 5. **(a)** Gene sets found enriched in the list of 911 genes with differing CN-alterations in the metastatic primary tumors (see methods) using GO enrichment analysis tool (http://geneontology.org/docs/go-enrichment-analysis/). Only those gene sets with fold-change enrichment (FCe)>1.8 and FDR<0.05 by Fisher's Exact test were considered. Column length indicates FCe as specified in the top horizontal-axis; black dots show $-\log_{10}$(FDR) as detailed in the bottom horizontal-axis. **(b)** GSEA analysis of gene sets appeared in (A) using RNA-Seq series of metastatic *versus* non-metastatic primary tumors. Column length shows the normalized enrichment score (NES) as quantified in the top horizontal-axis; black dots display $-\log_{10}$(FDR) as indicated in the bottom horizontal-axis. The 120 gene sets identified are listed in the left of panels (A) and (B); the letters' font is related to the annotated data repository used (GO Biological Process, GO Molecular Function, GO Cellular Component or Reactome pathways) as indicated in the legend. The terms related to 'translation and ribosomes' are shown in blue, those related to 'DNA repair and TP53 pathway' in green, those involved in 'splicing' in grey, those associated with 'cell cycle and chromatin regulation' in pink, and those related to 'ubiquitin and proteosome' in brown.

Supplementary Fig. 10

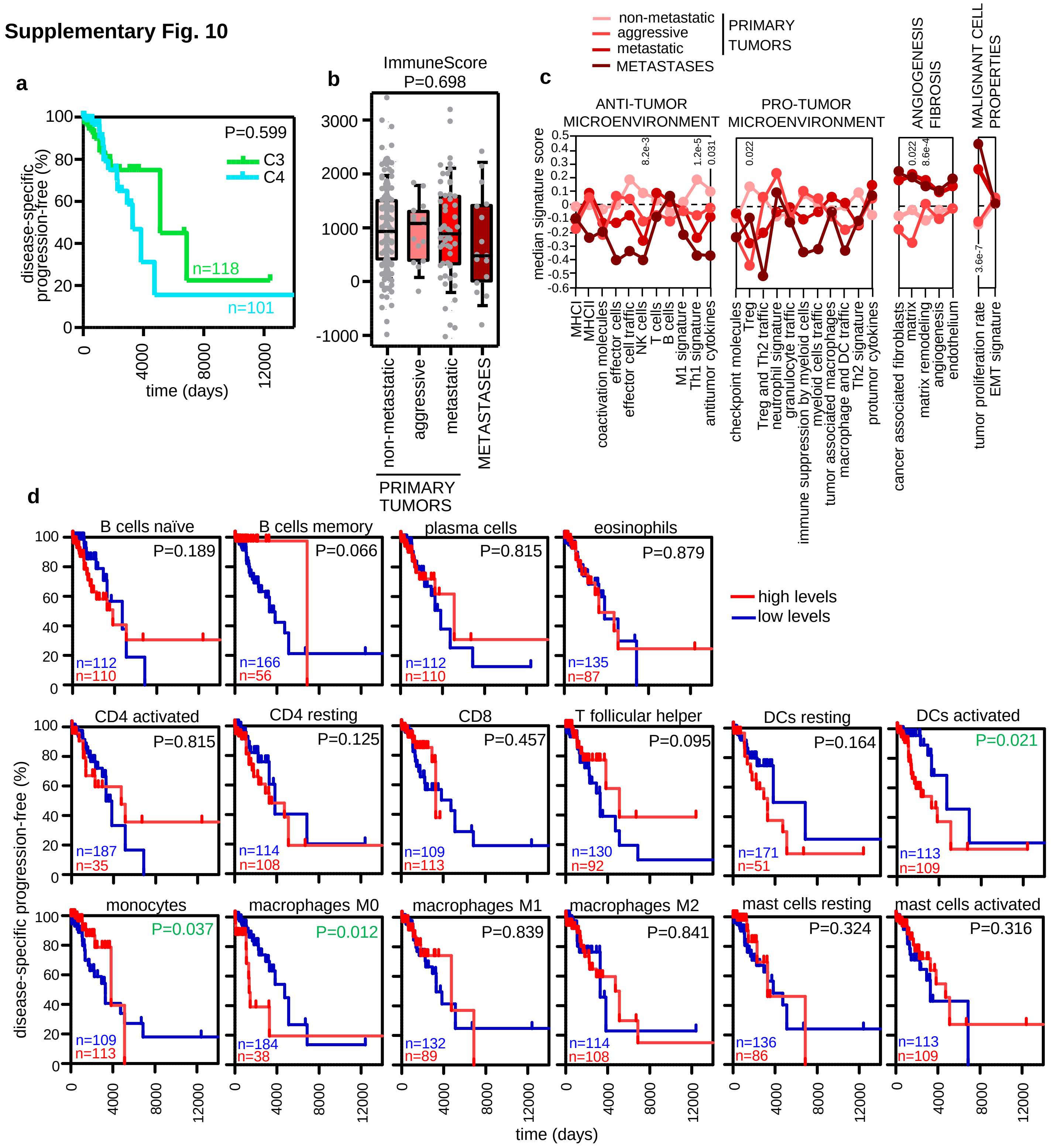

Supplementary Fig. 10. Immune landscape in PPGLs (II). (a) Progression-free survival analysis of patients according to immune subtype (n=118 for C3 and n=101 for C4). Kaplan-Meier plot of time to progression (time between the first PPGL diagnosis and the first documented metastases) is shown together with the P-value shown inside the plot was calculated using a log-rank test. Patients without evidence of metastases were censored at the date of the last follow-up. Only data from primary tumors was used to distinguish patients between clusters. Patients with tumors within C5 were not included in this analysis due to the low number of samples. **(b)** Box-plots depicting the ImmuneScore in the different tumor tissue types (n=262). Two-sided KW test was used to test for differences. The median value is marked, and Tukey whiskers are represented. **(c)** Median gene enrichment score of the different Fges in non-metastatic, aggressive, metastatic primary tumors, and metastases. KW was applied to test for differences between groups; P-value is shown. **(d)** Relative to Fig. 6e. Kaplan-Meier plots of time to progression in patients according to primary tumors immune cell type levels. Only primary tumors from non-metastatic and metastatic patients included. High levels (above the median level of the whole group) of the cell type is represented in red (n=110 for B cells naïve, n=56 for B cells memory, n=110 for plasma cells, n=87 for eosinophils, n=35 for CD4 activated, n=108 for CD4 resting, n=113 for CD8, n=92 for T follicular helper, n=113 for monocytes, n=38 for macrophages M0, n=89 for macrophages M1, n=108 for macrophages M2, n=51 for DCs resting, n=109 for DCs activated, n=89 for mast cells resting and n=109 for mast cells activated) and low expression (below the median level) in blue (n=112 for B cells naïve, n=166 for B cells memory, n=112 for plasma cells, n=135 for eosinophils, n=187 for CD4 activated, n=114 for CD4 resting, n=109 for CD8, n=130 for T follicular helper, n=109 for monocytes, n=184 for macrophages M0, n=132 for macrophages M1, n=114 for macrophages M2, n=171 for DCs resting, n=113 for DCs activated, n=136 for mast cells resting and n=113 for mast cells activated) . P-values shown inside the plots were calculated using a log-rank test. Patients without evidence of metastases were censored at the date of the last follow-up. n: number of patients.

Supplementary Fig. 11

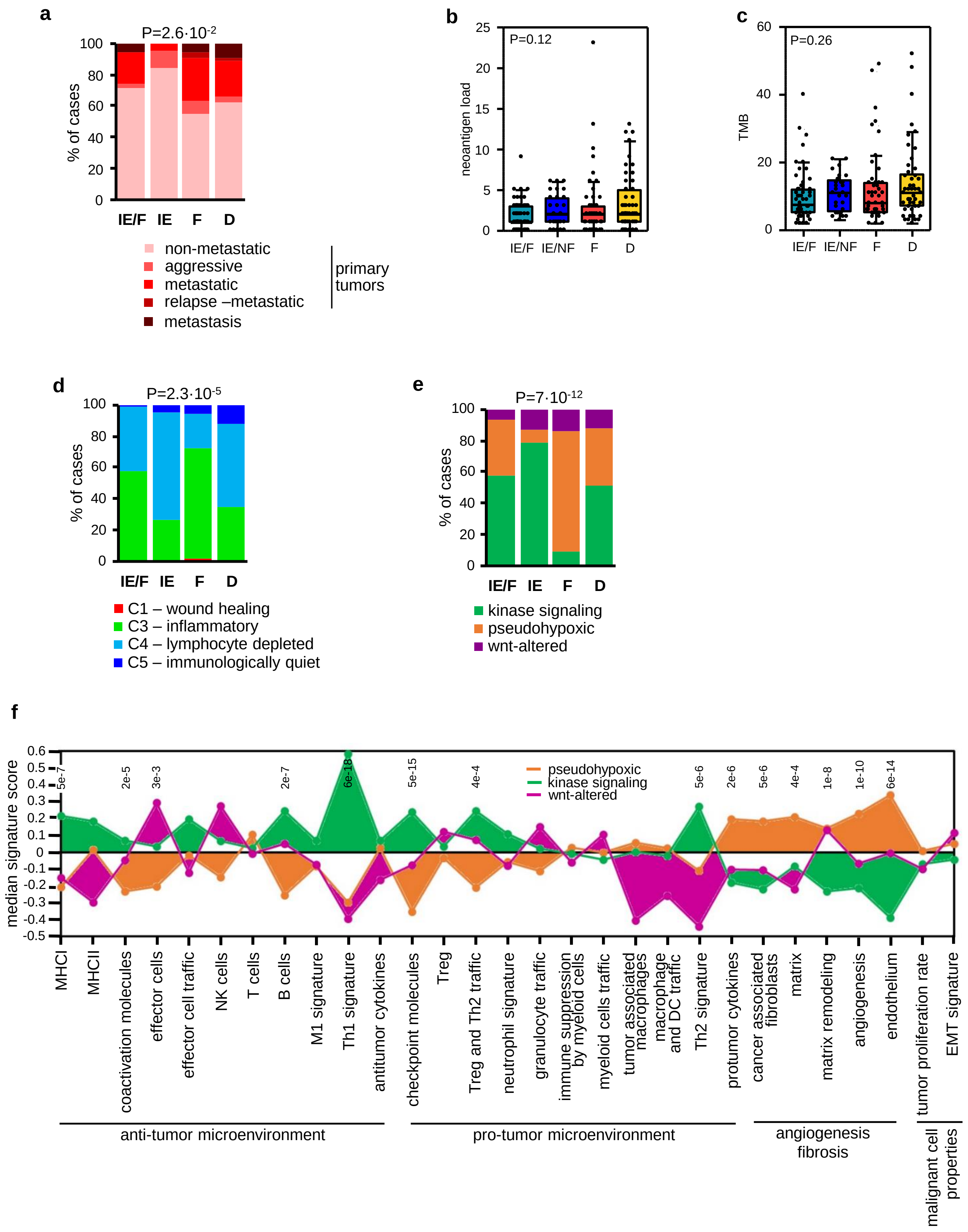

Supplementary Fig. 11. **Immune landscape in PPGLs (III)**. Percentage of tumors per TME subtype according to their **(a)** clinical behavior, **(d)** immune subtype or **(e)** genomic subtype. P-values were calculated using a two-sided Chi-squared test. **(b)** Neoantigen load and **(c)** TMB across the different TME subtypes in primary PPGL tumors with WES and RNA-Seq data available (n=180). The median value is marked, and Tukey whiskers are represented. P shown in the figure corresponds to a two-sided Krustal-Wallis test. **(f)** Median gene signature score of the different Fges in primary tumors according to their genomic subtype. Two-sided KW was applied to test for differences between groups; significant p-values are shown.

Supplementary Fig. 12

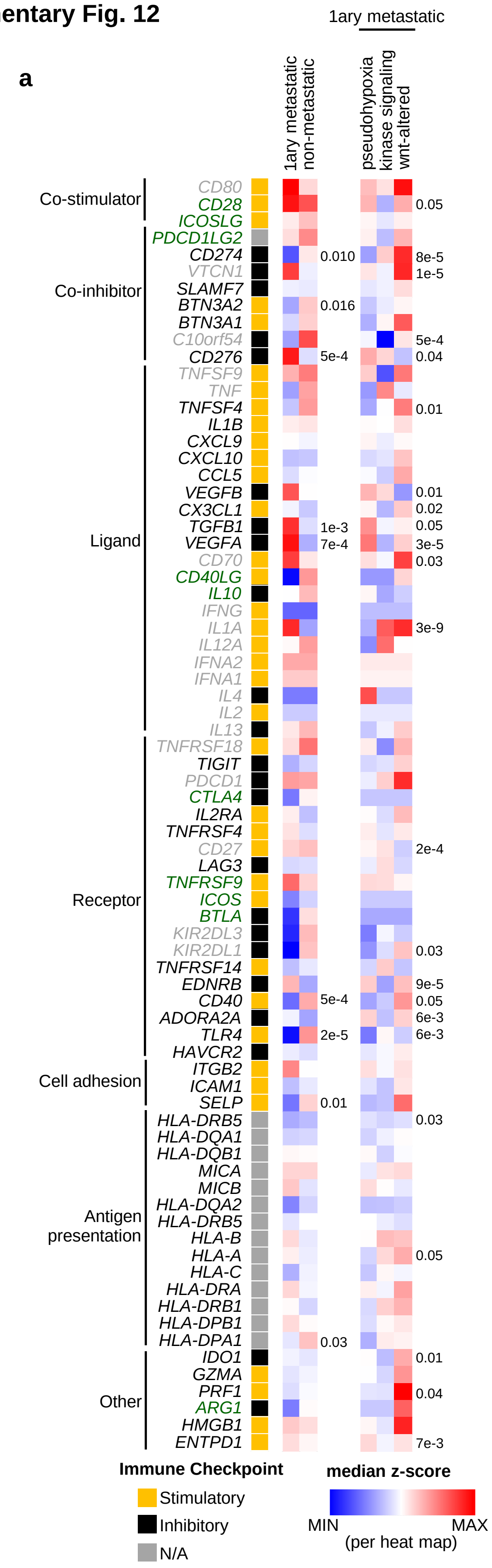

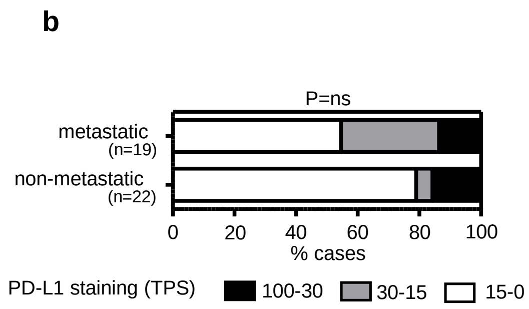

Supplementary Fig. 12. Immunogenomics as a theranostic tool in the immunotherapy contexture (II). (a) Expression (median normalized z-score expression) of 75 immunoregulators selected by Thorsson *et al*. In black, those included in Fig. 7c. Those in grey and green were not included due to unavailability of expression levels for all the tumors of the series: in grey, genes whose levels were only available for the TCGA series; and in green, genes which were discarded in a previous QC steps to obtaining the final matrix of both cohorts. Expression is shown according to the clinical behavior and genomic subtype. Two-sided MWW was applied to test for between metastatic and non-metastatic primary tumors; KW was used to test for differences between the different genomic subtypes; significant p-values are shown in the figure **(b)** Quantification of PD-L1 IHC staining in a subset of n=41 PPGLs. Quantification is represented within tumor behavior. Three PPGLs classified as aggressive were omitted from the analysis. Two-sided Freeman-Halton test was used to test for differences between groups.

Supplementary Fig. 13

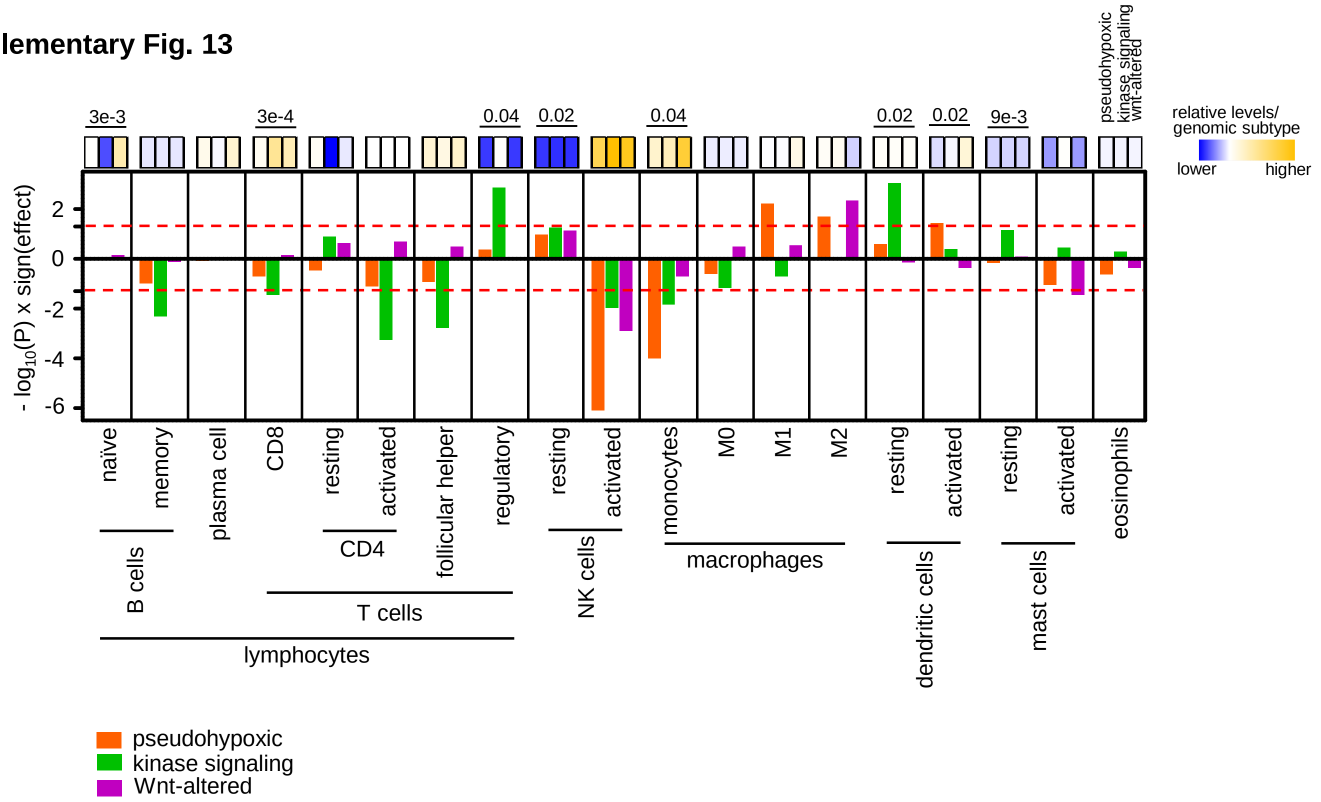

Supplementary Fig. 13. Composition of the tumor immune infiltrate. Related to Fig. 6b. Top: relative levels of the different immune cell classes estimated with CIBERSORTx (blue-lower levels, yellow-higher levels) by genomic subtype (in the following order: pseudohypoxic, kinase signaling and Wnt-altered). Two-sided Krustal-Wallis test (KW) was used to test for differences between the different genomic subtypes; significant p-values are shown in the figure. Bottom: Plot showing the $-log_{10}(P)$ resulting from a MWW analysis to define differences between metastatic primary *versus* non-metastatic tumors in the different immune cell classes by genomic subtype. The 'sign(effect)' indicates the direction of the fold-change between the proportions in both groups. Columns that surpass the red dashed line indicates $P<0.05$. Cell types with >85% of the samples with '0s' were excluded from the analysis.

Supplementary Table 3. Clinical characteristics of patients with pheochromocytomas and paragangliomas included in FFPE series.

**Patient's Clinical Characteristics**	**FFPE series (n = 44)** % (n)
**Primary Tumor Type**	
PCC	56.8% (25)
PGL	40.9% (18)
NA	2.3% (1)
**Sex**	
Female	45.4% (20)
Male	54.5% (24)
**Age at initial diagnosis of PCC/PGL; median (range) in years**	50 (15-82)
**Clinical behavior**	
Non-metastatic disease	50% (22)
Aggressive disease	6.8% (3)
Metastatic disease	43.2% (19)

Supplementary Table 4. Variant impact consequence classification

**Variant classification***	**VEP annotation**	**PolyPhen/SIFT/CONDEL predictions and COSMIC FATHMM prediction (if any)**
**LOW impact**	splice_region_variant	-
	start_retained_variant	-
	stop_retained_variant	-
	synonymous_variant	-
	missense_variant	benign/tolerated/neutral by all in silico predictors and neutral by COSMIC
**MODERATE impact**	protein_altering_variant	-
	inframe_deletion	-
	inframe_insertion	-
	missense_variant	at least one predictor benign/tolerated/neutral
**HIGH impact**	splice_acceptor_variant	-
	splice_donor_variant	-
	stop_gained	-
	frameshift_variant	-
	stop_lost	-
	missense_variant	all predictors as damaging/ deleterious/ deleterious
	all annotations	pathogenic by COSMIC

* as in Calculated variant consequences by Ensembl Variation (https://m.ensembl.org/info/genome/variation/prediction/predicted_data.html)

Supplementary Fig. 14

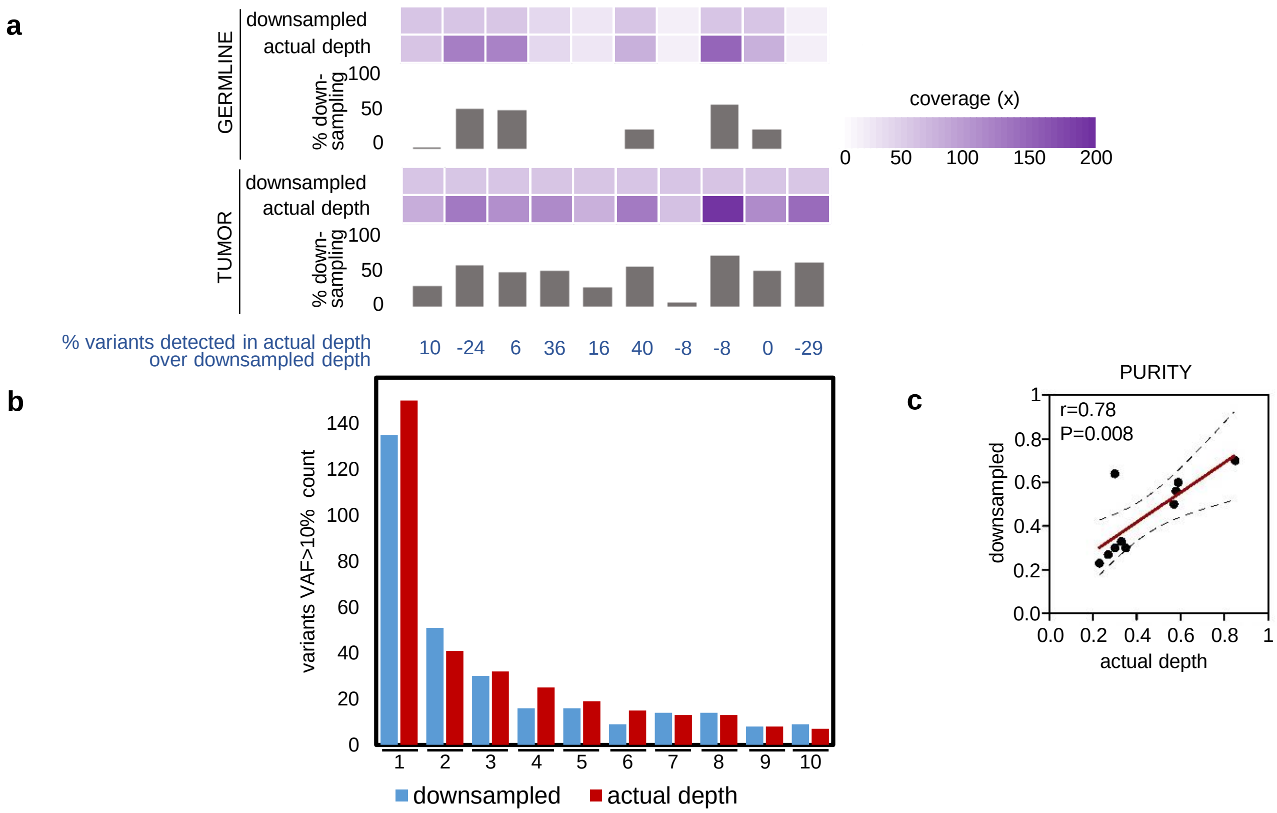

Supplementary Fig. 14. Effect of sequencing depth on variant calling. (a) Actual and downsampled depth (to resemble the median coverage of the TCGA cohort) of ten randomly selected samples. Comparison of variant calling count **(b)** and purity **(c)** in the selected samples. VAF: variant allele frequency. In c, the R squared (r), the P-value and the 95% confidence band of the best-fit line resulting from a linear regression analysis are shown.
