# Supplementary material for: Genomic and immune landscape Of metastatic pheochromocytoma and paraganglioma
Source: Nat Commun. 2023 Feb 28;14:1122. doi: 10.1038/s41467-023-36769-6 (PMC9975198; doi:10.1038/s41467-023-36769-6)
Supplement: Supplementary file 3 — Description of Additional Supplementary Files [file 41467_2023_36769_MOESM3_ESM.pdf]

Description of Additional Supplementary Files

File Name: Supplementary Data 1
Description: Receiver operating characteristic curve analysis showing the classification power of different markers of mPPGL. AUC, 95% confidence interval (CI) and sensitivity/specificity per each of the 511 classifiers.
