# Supplementary material for: Genomic and immune landscape Of metastatic pheochromocytoma and paraganglioma
Source: Nat Commun. 2023 Feb 28;14:1122. doi: 10.1038/s41467-023-36769-6 (PMC9975198; doi:10.1038/s41467-023-36769-6)
Supplement: Supplementary file 5 — Reporting Summary [file 41467_2023_36769_MOESM5_ESM.pdf]

nature portfolio

Corresponding author(s): Bruna Calsina
Mercedes Robledo

Last updated by author(s): Feb 3, 2023

Reporting Summary

Nature Portfolio wishes to improve the reproducibility of the work that we publish. This form provides structure for consistency and transparency in reporting. For further information on Nature Portfolio policies, see our Editorial Policies and the Editorial Policy Checklist.

Statistics

For all statistical analyses, confirm that the following items are present in the figure legend, table legend, main text, or Methods section.

n/a	Confirmed	
☐	☒	The exact sample size (*n*) for each experimental group/condition, given as a discrete number and unit of measurement
☐	☒	A statement on whether measurements were taken from distinct samples or whether the same sample was measured repeatedly
☐	☒	The statistical test(s) used AND whether they are one- or two-sided<br>*Only common tests should be described solely by name; describe more complex techniques in the Methods section.*
☐	☒	A description of all covariates tested
☐	☒	A description of any assumptions or corrections, such as tests of normality and adjustment for multiple comparisons
☐	☒	A full description of the statistical parameters including central tendency (e.g. means) or other basic estimates (e.g. regression coefficient) AND variation (e.g. standard deviation) or associated estimates of uncertainty (e.g. confidence intervals)
☐	☒	For null hypothesis testing, the test statistic (e.g. *F*, *t*, *r*) with confidence intervals, effect sizes, degrees of freedom and *P* value noted<br>*Give P values as exact values whenever suitable.*
☒	☐	For Bayesian analysis, information on the choice of priors and Markov chain Monte Carlo settings
☐	☒	For hierarchical and complex designs, identification of the appropriate level for tests and full reporting of outcomes
☐	☒	Estimates of effect sizes (e.g. Cohen's *d*, Pearson's *r*), indicating how they were calculated

Our web collection on statistics for biologists contains articles on many of the points above.

Software and code

Policy information about availability of computer code

Data collection	Whole exome sequencing (WES) libraries were prepared with the SureSelectXT Human All Exon V6+COSMIC target enrichment system (Agilent, 5190-9307) following the manufacturer`s instructions. Sequencing was performed on either the Illumina HiSeq2500 or NovaSeq6000 in a paired-end 100bp reads mode to a median target coverage of ~200x and ~100x for tumors and germline, respectively.<br>RNA library preparation with RNA Integrity Number (RIN) > 5.5 was performed as described in the TruSeq Stranded mRNA Library Prep Kit (Illumina, RS-122-2101) and sequenced on an Illumina HiSeq2500 on a 51bp single-read format following manufacturer's protocols.<br>cDNA libraries from FFPE tumors and low integrity RNA's (RIN<5.5) were prepared using QuantSeq 3' mRNA-Seq Library Prep Kit FWD for Illumina (Lexogen, 015) with a UMI Second Strand Synthesis Module for QuantSeq FWD (Lexogen, 081). Libraries were applied to an Illumina flow cell for cluster generation and sequenced on NovaSeq6000.
Data analysis	The following pipelines were used:<br>Illumina Real Time Analysis software<br>bcl2fastq Software (Illumina)<br>Burrows-Wheeler Aligner (BWA) (ref 80)<br>Genome Analysis Toolkit GATK (ref 81)<br>Haplotype Caller (ref 82)<br>MuTect (ref 83)<br>FACETS (ref 85)<br>MANTIS (ref 86)<br>VEP (ref 84) v106

biomaRt (ref 88)
GISTIC 2.0 (ref 90)
bcl2fastq software (Illumina)
Nextpresso (ref 92)
BlueBee Genomics Platform (Lexogen)
TopHat v2.1.1 (ref 93)
STAR v2.5.2a (ref 94)
ESTIMATE v1.0.13 (ref 36)
Combat (ref 95)
R/Bioconductor sva v3.26.0 (ref 96)
DESeq2 v1.18.1 (ref 97)
String v11 (https://string-db.org/) (ref 21)
GSEA v2.2.2 (ref 99)
Panther (ref 25) 17.0
CIBERSORTx (ref 37)
ImmuneSubtypeClassifier of the Cri-iATLAS v0.1.0 (ref 100)
GSVA (ref 101)
Morpheus (https://software.broadinstitute.org/morpheus)
pVACtools (ref 102) v3.0.2
bam-readcount (Larson. genome/bam-readcount. GitHub https://github.com/genome/bam-readcount)
Docker 1.1.1 image of mgibio/bam_readcount_helper-cwl
vcf-readcount-annotator from the VAtools package (http://vatools.org)
polysolver (ref 103) v4
Zen Blue software (Zeiss)
QuPath (ref 104)
R v3.2.2 and v4.0.3
IBM SPSS Statistics v19

For manuscripts utilizing custom algorithms or software that are central to the research but not yet described in published literature, software must be made available to editors and reviewers. We strongly encourage code deposition in a community repository (e.g. GitHub). See the Nature Portfolio guidelines for submitting code & software for further information.

Data

Policy information about availability of data

All manuscripts must include a data availability statement. This statement should provide the following information, where applicable:

- Accession codes, unique identifiers, or web links for publicly available datasets
- A description of any restrictions on data availability
- For clinical datasets or third party data, please ensure that the statement adheres to our policy

The WES and RNA-Seq fasq files generated during this study, as well as level 3 files (expression count matrix, and the VCF files with the variant calling results for both SNV/INDELs and CN) have been deposited in the European Genome-Phenome Archive (EGA) under the accession EGAS00001006043 and EGAS00001006044. The data are available under restricted access due to the possibility of revealing patient-sensitive information. Request for data access will be referred di-rectly to the Data Access Committee (DAC) of the CNIO (mrobledo@cnio.es). The access will be granted for health/medical/biomedical purposes and according to good practice recommenda-tions. The DAC will attempt to provide a response to all applications within two weeks of submission and render a final decision within no more than four weeks. Once access has been granted, data will be available during one month. The remaining data are available within the article and supplementary information. Source Data required for the reproduction of figures presented in this study a. The public-ly available microarray dataset used in this study is available from ArrayExpress (E-MTAB-733)(ref20) and the TCGA WES and RNA-Seq data from GDC Data Portal, NIH (https://portal.gdc.cancer.gov/), TCGA-PCPG(ref5).

Human research participants

Policy information about studies involving human research participants and Sex and Gender in Research.

Reporting on sex and gender	Sex was taken into account as a co-variate in some analyses as indicated either in the ms text or in the figure caption. Both sexs were represented by 40-55% in the WES, RNA-Seq and IHC cohorts. Gender data was not collected for this study.
Population characteristics	Patient's cohort used for WES series included 20 patients with PCC, 19 with PGL, 2 with bilateral PCC, 7 with multiple PGLs, 8 with PCC and PGL, and 5 with unknown location of the tumor. From these, 2 had non-metastatic disease, 3 had aggressive disease, and 56 had metastatic disease.<br>Patient's cohort for RNA-Seq analysis included 39 patients with PCC, 38 with PGL, 4 with bilateral PCC, 4 with multiple PGL, 11 with PCC and PGL, and 8 with unknown location of the tumor. From these, 43 had non-metastatic disease, 10 aggressive disease, and 51 metastatic disease.<br>Patient's cohort for the FFPE series included 25 patients with PCC, 18 with PGL and 1 with unknown location of the tumor. From these, 22 had non-metastatic disease, 3 had aggressive disease and 19 metastatic disease.<br>Genotype and/or genomic subtype and sex were taken into account in analyses as indicated as co-variates
Recruitment	Patients were recuited to tissue banks under institutional ethical-approved protocols who underwent surgery for the relevant cancer type. A total of 73 patients were enrolled between January 2017 and April 2019 and included in WES study. For the

	RNA-Seq study, 165 patients were enrolled between January 2017 and August 2019. The cohort is composed of consecutive samples from metastatic patients, without any other bias selection.
Ethics oversight	The study was conducted in accordance with the Declaration of Helsinki, and the protocol was approved by the following Ethics Committees: Hospital Universitario 12 de Octubre (15/024), Madrid, Spain; Universitäts Spital Zurich (2017-00771), Zurich, Switzerland; Klinikum der Universität (379-10), Munich, Germany; University of Würzburg (88/11), Würzburg, Germany; Azienda Ospedaliera Universitaria Careggi (Prot. N. 2011/0020149), Florence, Italy; Berlin Chamber of Physicians (Eth-S-R/14), Berlin, Germany; Radboud University Medical Centre (9803-0060), Nijmegen, The Netherlands; TU Dresden (EK210052017 and EK189062010), Dresden, Germany.

Note that full information on the approval of the study protocol must also be provided in the manuscript.

Field-specific reporting

Please select the one below that is the best fit for your research. If you are not sure, read the appropriate sections before making your selection.

[x] Life sciences [ ] Behavioural & social sciences [ ] Ecological, evolutionary & environmental sciences

For a reference copy of the document with all sections, see nature.com/documents/nr-reporting-summary-flat.pdf

Life sciences study design

All studies must disclose on these points even when the disclosure is negative.

Sample size	No statistical method for sample size determination was performed. We performed genomic profiling of 156 PPGLs from 128 unrelated patients. This is an observational exploratory study reporting descriptive data. The size of the cohort was arbitriary determined by the availability of patient's samples. Given the clear nature of the findings and the number of metastatic cases found in the population (1 in 1 milion), we believe that the cohort size is outstanding.
Data exclusions	For the WES series, excusion criteria was as follows: (i) no germline available, (ii) DNA quality control failure, (iii) WES analysis quality control failure, (iv) tumor and germline did not pair according to NGSCheckMate, (v) tumor content purity lower than 12% according to FACETS. For the RNA-Seq series, samples failing quality control (e.g. low number of reads) or with estimate score greater than 5900 were excluded. Also, samples considered outliers according to PCA, and consensus and hierarchical clustering were removed from further analysis.
Replication	No technical replication was performed for WES, RNA-Seq or IHC experiments. Immune profile and the CDK1 levels were validated using IHC. All other findings were not technically replicated due to limited sample material. The gene signature identified was replicated in one publically available independent cohort as described in the ms.
Randomization	Samples were randomised before generation of WES and RNA-Seq data.
Blinding	Blinding of samples was performed for data collection. No blinding of samples was done during analysis as knowing the samples characteristics was needed to assign them to specific groups.

Reporting for specific materials, systems and methods

We require information from authors about some types of materials, experimental systems and methods used in many studies. Here, indicate whether each material, system or method listed is relevant to your study. If you are not sure if a list item applies to your research, read the appropriate section before selecting a response.

Materials & experimental systems

n/a	Involved in the study	
[ ]	[x]	Antibodies
[x]	[ ]	Eukaryotic cell lines
[x]	[ ]	Palaeontology and archaeology
[x]	[ ]	Animals and other organisms
[x]	[ ]	Clinical data
[x]	[ ]	Dual use research of concern

Methods

n/a	Involved in the study	
[x]	[ ]	ChIP-seq
[x]	[ ]	Flow cytometry
[x]	[ ]	MRI-based neuroimaging

Antibodies

Antibodies used	mouse anti-CDK1 Ab (dil 1:250; BD Biosciences Cat# 610038, RRID:AB_397454)<br>rabbit anti-PDL1 [E1L3N] XP mAb (dil 1:150 20'ER2; Cell Signaling Technology Cat# 13684, RRID:AB_2687655)<br>anti-CD8A [NOR132H] (dil 1:20; CNIO in-house Ab)
Validation	anti-CDK1 Ab and anti-PDL1 are validated for the daily routine diagnostic workflow in the Histophatology Unit at the CNIO.

Anti-PDL1 has also been validated for IHC analysis of FFPE samples of human lung, breast and ovarian carcinoma, human placenta, and Karpas-299 and PC-3 cell lines. It has been cited over 830 times. Anti-CDK1 validation for IHC at the CNIO was firstly publish by Garcia JF et al. Blood. 2003; 101(2):681-689 (PMID: 12393683).
Anti-CD8A is CNIO in-house Ab validated by EuroMAbNet; https://www.euromabnet.com/monoclonal-antibodies/cd8a/38.html
